# Readiness and Acceptance of Nursing Students Regarding AI-Based Health Care Technology on the Training of Nursing Skills in Saudi Arabia: Cross-Sectional Study

**DOI:** 10.2196/71653

**Published:** 2025-07-30

**Authors:** Kamlah AL-Olaimat, Basma Salameh, Rasha Abdulhalim Alqadi, Abeer Alruwaili, Manal Hakami, Hanay Huwaydi ALanazi, Tahani Maharem, Fadia Ahmed Abdelkader Reshia

**Affiliations:** 1 Obstetrics and Gynecology Nursing, Faculty of Nursing, Zarqa University, Zarqa, Jordan; 2 Nursing Department, Faculty of Nursing, Arab American University, P.O Box 240 Jenin, 13 Zababdeh, Jenin, 00972, Palestinian Territory, Occupied, 970 0569357783; 3 Medical-Surgical Nursing Department, North Private College of Nursing, Arar, Saudi Arabia; 4 Nursing Administration and Education Department, College of Nursing, Jouf University, Sakakah, Saudi Arabia; 5 Medical Surgical Nursing Department, Faculty of Nursing, Al-Baha University Al Baha Saudi Arabia, Al Bahah, Saudi Arabia; 6 Medical Surgical Nursing Department, Alexandria University, Alexandria, Egypt; 7 Critical Care and Emergency Nursing Department, Faculty of Nursing, Mansoura University, Mansoura, Egypt; 8 Medical Surgical Nursing, College of Nursing, Jouf University, Sakaka, Saudi Arabia

**Keywords:** artificial intelligence, technology, readiness, acceptance

## Abstract

**Background:**

The rapid advancements in artificial intelligence (AI) technologies across various sectors, including health care, necessitate the need for a comprehensive understanding of their applications. Specifically, the acceptance and readiness of nursing students as future health care professionals to adopt AI-based health care technologies, along with the factors influencing these attitudes, are critical for facilitating the effective integration of AI in health care settings.

**Objective:**

This study aimed to assess the readiness and acceptance of nursing students regarding the use of AI-based health care technologies in the nursing skills training in Saudi Arabia.

**Methods:**

A descriptive cross-sectional research design was used. A convenience sampling technique was applied to recruit 322 participants. Data were collected between June and September 2023 using a self-administered questionnaire that included the technology readiness index (TRI) and the technology acceptance scale.

**Results:**

Approximately 92.2% (297/322) of participants exhibited positive attitudes toward AI, and 74.8% (241/322) demonstrated innovativeness, indicating a generally favorable perception of AI. However, more than half of the students (59% [190/322] and 59.3% [191/322], respectively) reported feelings of discomfort and negative perceptions regarding AI use. Regarding TRI, 69.6% (224/322) of participants showed moderate readiness, while 30.4% (98/322) exhibited a high level of TRI. A substantial majority (320/322 99.4%) expressed acceptance of AI-based technologies in their training, with only 0.6% (2/322) reporting nonacceptance. Older students (aged >22 y) exhibited significantly higher levels of AI acceptance and readiness compared to younger students (*P*<.001). In addition, female students demonstrated significantly greater readiness and acceptance levels than male students (*P*=.003). Further, third-level students reported the highest mean scores in both acceptance and readiness (66.77 and 16.69, respectively; *P*=.002), while first-level students had the lowest (60.59 and 15.15). Among course groups, students enrolled in Maternal and Child Health Nursing reported the highest mean scores (65.19 and 16.30), whereas those in Community Health Nursing reported the lowest (57.50 and 14.38; *P*<.001).

**Conclusions:**

The findings indicate that nursing students demonstrated a generally positive level of readiness and acceptance toward the use of AI and related technologies in education and training. However, these levels remained moderate overall, highlighting the need to enhance awareness and deepen students’ understanding of AI’s potential to improve training effectiveness and health care quality.

## Introduction

Artificial intelligence (AI) refers to the development of computer systems capable of performing tasks that typically require human intelligence. AI is currently one of the most transformative forces globally, with a particularly significant impact on the health care sector. It holds the potential to revolutionize various aspects of health care, from hospital operations and clinical trials to pharmaceutical development. These applications aim to improve health care delivery and reduce costs for both institutions and patients [1]. In this context, readiness refers to individuals’ preparedness to adopt and effectively use new technologies. In the context of education, the emergence of new behavioral change is closely linked to the student’s level of readiness. Readiness plays a critical role in enabling nursing students to adapt to future advancements in health care technologies. It is particularly essential for the effective integration of AI into nursing education and clinical practice, as it equips students to navigate a technology-driven health care environment. Therefore, assessing nursing students’ readiness and attitudes is important for the successful integration of AI into their future professional roles [2].

The AI market in health care, valued at US $15.4 billion in 2022, is projected to grow at a compound annual growth rate of 37.5% from 2023 to 2030. This rapid expansion is driven by several key factors, including the increasing volume of digital patient health data, a rising demand for personalized treatment, and the need to reduce health care costs [3]. Over the past 5 years, the impact of AI technologies on nurse educators, nursing students, and practicing nurses has been the focus of extensive research, highlighting the transformative potential of AI in nursing education and clinical practice [4,5]. To effectively disseminate knowledge about AI algorithms and their applications, which are expected to play a significant role in both medical and nursing fields, it is recommended that medical and nursing schools integrate AI into their curricula [6-8]. A systematic review and meta-analysis by Amiri et al [9] examined the attitudes and knowledge of medical, dental, and nursing students toward AI in health care. The findings revealed a moderate level of knowledge (3736/8491, 44%) and a generally positive attitude (5519/8491, 65%), suggesting a promising level of acceptance of AI technologies among health care students.

In situations where clinical coaching opportunities are limited, the integration of innovative technologies significantly enhances nursing education by supporting professional skill development and effective training [10,11]. Nurse educators must become both knowledgeable and confident in the use of emerging AI health technologies. In addition, practicing nurses require an urgent need for upskilling to effectively integrate AI health technologies into clinical practice [12]. While technological advancements in nursing have been substantial, the impact of AI is particularly transformative. Examples include medication-dispensing robots, companion robots for individuals with special needs, and AI systems used for population health management and care coordination [13]. Registered nurses must understand the role of AI in modern health care delivery. Therefore, nursing education must clearly define the competencies required for integrating AI into practice to ensure its optimal use and value [14].

An Egyptian study conducted at Cairo University revealed that fourth-year nursing students held moderate attitudes toward AI [15]. A systematic mapping review of 22 articles indicated that virtual reality simulations are used for diverse educational purposes, including procedural skills training, emergency response training, and psychomotor skills development [16]. In addition, a scoping review of 131 papers on AI technologies showed that AI is already impacting nursing roles, workflows, and nurse-patient interactions, suggesting that AI-powered solutions have the potential to improve nursing practice. However, nurses must proactively ensure that person-centered, compassionate care remains in the integration of AI [17].

Student nurses’ attitudes and intentions regarding AI use are influenced by their perceptions of its role in nursing practice [18]. To successfully integrate AI into clinical settings, it is essential to understand nurses’ attitudes and behaviors toward both current and anticipated AI applications.

Evaluating their current level of AI knowledge is crucial to identify future training needs [19]. Although nursing attitudes significantly affect AI acceptability and adoption, research in this domain remains limited [18]. Thus, this study aims to assess the readiness and acceptance of nursing students in Saudi Arabia regarding the use of AI in the nursing skills training. This investigation seeks to provide valuable insights into the factors that influence nursing students’ preparedness and acceptance to embrace AI in their education. Despite a growing global interest in integration of AI in health care, there remains a significant knowledge gap in Saudi Arabia, where only a limited number of studies have explored this area. Addressing this gap is essential to effectively align health care education within international best practices and emerging technological trends. Given the global shift toward integrating AI into both health care and nursing education, it is particularly important to understand nursing students’ readiness and acceptance of this technology. Ultimately, this study will contribute to ensuring that future nursing graduates are equipped to adopt AI technologies in clinical settings, thereby enhancing the quality of patient care in line with international standards.

## Methods

### Study Design and Settings

This study used a descriptive cross-sectional research design and was conducted at 3 universities in Saudi Arabia among undergraduate nursing students. Data collection took place between June and September of the 2023 academic year.

To ensure accessibility, data were collected using a self-administered online survey developed in Arabic via Google Forms. The survey link was disseminated through various social media platforms, including WhatsApp (Meta), Instagram (Meta), and Facebook (Meta), by coinvestigators at each participating university. Participants were instructed to complete only one version of the survey to maintain data integrity and prevent duplicate responses.

### Sample Size

A convenience sampling technique was used to collect the data from a total of 322 undergraduate nursing students enrolled in 3 Saudi universities. These universities were initially selected using a multistage probability sampling technique. The required sample size was calculated using the open-source calculator OpenEpi, Version 7, based on a total population of 1983 nursing students. The sample size calculation was based on an expected frequency of 50%, a 5% margin of error, and a 95% confidence level, resulting in a minimum sample size of 322 students. Proportional allocation was used to determine the number of participants from each university: (100 students from Al-Baha University (population =618), 44 students from Al-Jouf University (population =265), and 178 students from Al-Shamal Faculty of Nursing (population =1100).

### Tool of the Study

The questionnaires used for data collection were divided into 3 sections. Section one collected sociodemographic data from nursing students, including age, gender, academic level, and specialty training course. Section 2 consisted of the Technology Readiness Index (TRI), a tool originally developed by Parasuraman and Colby [20] and adapted for this study. This section included 16 items designed to measure technology readiness across four dimensions: for optimism (4 items), innovativeness (4 items), discomfort (4 items), and insecurity (4 items). Each item was rated on a 5-point Likert scale ranging from 1 (strongly disagree) to 5 (strongly agree). Optimism and innovativeness reflect positive predispositions toward technology, while discomfort and insecurity represent negative tendencies. To calculate the overall total TRI Score, the negatively worded dimensions (insecurity and discomfort) were reverse coded by subtracting each item score from 6. The final TRI score was computed using the formula: TRI = (Innovative+Optimism+(6-Insecurity) + (6-Discomfort))÷4). The score ranges from 1.0 to 5.0, with higher scores indicating greater technology readiness. For interpretation, readiness levels were categorized as follows: low (<2.0), moderate (<3.5), and high (>3.5).

Section three focused on the technology acceptance model (TAM), originally developed by Davis [21] and adapted by the researcher of this study. This section included 16 items grouped into 3 key constructs to measure technology acceptance: Perceived usefulness (6 items), perceived ease of use (6 items), and intention to use (4 items). All items were rated on a 5-point Likert scale ranging from 1 = extremely unlikely to 5 = extremely likely. To calculate the average perception score, the total score of all TAM items was summed for each participant. This raw score was then converted into a percentage by dividing it by the maximum possible score and multiplying by 100. The average percentage was computed across all participants to determine the overall mean perception score. Perception levels were categorized as follows: low (<65%), moderate (65% to <80%), and high (≥80%). For interpretive purposes, scores <40% were considered not accepted, while scores >40% were considered accepted.

To ensure clarity for comprehension for all nursing students, the survey was initially translated into Arabic and then back-translated into English to verify accuracy and consistency. Content validity was evaluated by a panel of five experts, all holding PhDs in nursing. The experts were requested to evaluate the relevance and clarity of each item, provide suggestions for improvement, and determine whether the items appropriately reflected the objectives of the study. Reliability of the instruments was assessed using Cronbach α. For TRI, which consisted of 16 items, the reliability coefficient was *α* =. 820. For TAM, also comprising 16 items, the reliability coefficient was *α*=.782.

### Pilot Study

A pilot study was carried out on 10% of the study sample (N=32) to assess the clarity, feasibility, and applicability of the survey tools. Participants in the pilot study were excluded from the final sample to avoid bias. The average time to complete the survey ranged from 15 to 20 minutes. The study was conducted across three universities: Al-Baha University (n=618), Al-Jouf University (n=265), Al-Shamal Faculty of Nursing (n=1100).

### Ethical Considerations

Ethical approval for this study was obtained from the Research Ethics Committee, Faculty of Nursing, Al-Baha University (approval number 44127970). In addition, official permission letters were secured from the Faculty of Nursing in each sitting Al-Baha University, Al-Jouf University, and Al-Shamal Faculty of Nursing to conduct the study and collect data. The study was conducted in accordance with the ethical principles of the Declaration of Helsinki. Participation in the study was entirely voluntary, with no academic consequences for students who chose to participate. Anonymity and confidentiality were strictly maintained by not collecting names or any personally identifiable information. All data was securely stored and used exclusively for research proposals. Before participation, written informed consent was obtained from all participants.

There were no consequences or effects on grades for not participating; participation was completely voluntary. Participant anonymity was guaranteed by withholding names or any other personally identifying information. All data was safely stored and used solely for this study. Every participant gave their written informed consent.

### Statistical Analysis

Data were analyzed using IBM SPSS software, version 20.0. The Kolmogorov-Smirnov test was used to assess the normality of data distribution. Quantitative variables were summarized using range (minimum and maximum), mean, and SD. Statistical significance was determined at *P* value <.05. For inferential statistics, the student’s *t* test was used to compare between 2 independent groups for normally distributed data, while a one-way Analysis of Variance (*F* test) was applied to compare means among more than 2 groups. To identify which group differed significantly, post hoc analyses were conducted using the Tukey HSD test.

## Results

Table 1 presents the demographic characteristics of the study participants. The age of participants ranged from 18 years to over 22 years. Approximately half of students (154/322, 47.8%) were aged between 18 and 22 years, while a low percentage (49/322, 15.2%) were older than 22 years. Regarding gender, 47.2% (152/322) of the participants were male, and 52.8% (170/322) were female. In terms of academic level, the majority of students were in their second year (133/322, 41.3%) and third year (103/322, 32.0%). A smaller percentage was in their first year (27/322, 8.4%), while (59/322, 18.3%) were in their fourth year. In addition, nearly half of the participants (150/322, 46.6%) were enrolled in administrative nursing specialty training, while one quadrant (82/322, 25.5%) was in adult nursing specialty training. On the other hand, the lowest percentage (14/322, 4.3%) of participants was in Psychiatric nursing specialty training.

Table 2 illustrates the TRI findings among the nursing students. The majority of students reported high levels of optimism (297/322, 92.2%) and innovativeness (241/322, 74.8%), reflecting positive attitudes regarding the use of technology. Conversely, more than half of the participants reported moderate feelings of discomfort (190/322, 59.0%) and insecurity (191/322, 59.3%), indicating some negative feelings regarding technology. In addition, approximately 69.6% (224/322) of the participants reported moderate TRI, while 30.4% showed high TRI. Furthermore, the mean TRI score across all participants was 3.30 (SD =0.46), suggesting moderate levels of readiness to use technology in nursing skills training.

Table 3 presents participants’ levels of acceptance regarding various dimensions of technology use. A majority (187/322, 58.1%) exhibited high acceptance in terms of perceived usefulness of technology, while 29.2% (94/322) showed moderate acceptance and 12.7% (41/322) reported low acceptance in this domain. Regarding perceived ease of use, about half of the participants reported low acceptance, whereas 25.2% (81/322) and 18.6% (60/322) showed moderate and high acceptance, respectively. Concerning the intention to use or adapt the technology, 40.7% (131/322) showed high acceptance, while 34.8% (112/322) and 24.5% (79/322) reported moderate and low acceptance, respectively. Overall, 34.8% (112/322) of the participants demonstrated high technology acceptance, 40.1% (129/322) had moderate acceptance, and 25.2% (81/322) showed low acceptance.

reveals that the vast majority of students (320/322, 99.4%) reported overall acceptance of using technology, while only a small fraction (2/322, 0.6%) indicating no acceptance.

Table 4 compares nursing students’ acceptance and readiness to use AI and modern technology. The results indicate that students exhibited higher acceptance of technology and AI, with SD of 61.80±9.08 , 65.34 (SD 10.27), and 67.37 (SD 10.49), respectively, while their readiness scores were comparatively lower at (15.45 SD 2.27,16.33 SD 2.57, 16.84 SD 2.62). A significant difference was observed with respect to age: students older than 22 years showed a greater acceptance and readiness compared to younger students aged 18‐20 or 20–22 years, with statistical significance (*P*<.001). In addition, women show a high level of acceptance and preparedness to use technology and artificial intelligence, with a statistical significance of (0.003). The mean for females was (16.38 SD 2.38, 65.51 SD 9.50) compared to males (15.55 SD 2.55, 62.21 SD 10.21). Our findings reveal that third-level students exhibited the greatest acceptance and readiness (66.77 SD 10.04, 16.69 SD 2.51), while first-level students exhibited the lowest (60.59 SD 10.44, 15.15 SD 2.61), showing a statistical significance of (*P*=.002). Moreover, students participating in the Maternal and Child Health Nursing course exhibited the highest levels of acceptance and readiness (65.19 SD 9.41, 16.30 SD 2.35), while those in the Community Health Nursing course displayed the least acceptance (57.50 SD 7.93, 14.38 SD 1.98), with a statistical significance of (*P*<.001).

Turkey HSD Post hoc showed that students >20 years had significantly higher AI readiness and acceptance compared to those aged 18‐20. Third-year students had significantly greater AI readiness and acceptance than first- and second-year students. Students in the administrative nursing specialty showed significantly higher AI readiness and technology acceptance than those in other specialties.

**Table 1.** Distribution of the studied nursing students according to demographic data (n=322).

Demographic data	Values, n (%)
Age (years)	
18‐20	154 (47.8)
20‐22	119 (37.0)
>22	49 (15.2)
Sex	
Male	152 (47.2)
Female	170 (52.8)
Academic level	
First-year	27 (8.4)
Second year	133 (41.3)
Third year	103 (32.0)
Fourth year	59 (18.3)
Specialty	
Maternal and child nursing	26 (8.1)
Psychiatric nursing	14 (4.3)
Community nursing	26 (8.1)
Critical nursing	24 (7.5)
Administrative nursing	150 (46.6)
Adult nursing	82 (25.5)

**Table 2.** Distribution of the studied nursing students according to overall artificial intelligence readiness items (n=322).

Artificial intelligence readiness	Low (<2), n (%)	Moderate (2–3.5), n (%)	High (≥3.5), n (%)	Score range	Total score, mean (SD)	Average score (1–5), mean (SD)
Optimism	1 (0.3)	24 (7.5)	297 (92.2)	4–20	17.88 (2.63)	4.47 (0.66)
Innovativeness	3 (0.9)	78 (24.2)	241 (74.8)	4–20	16.02 (3.37)	4.01 (0.84)
Discomfort	80 (24.8)	190 (59)	52 (16.1)	4–20	9.81 (3.98)	2.45 (1.0)
Insecurity	100 (31.1)	191 (59.3)	31 (9.6)	4–20	9.08 (3.45)	2.27 (0.86)
Overall	0 (0)	224 (69.6)	98 (30.4)	16–80	15.99 (2.49)	3.30 (0.46)

**Table 3.** Distribution of the studied nursing students according to overall technology acceptance items (n=322).

Technology acceptance	Low (<65%), n (%)	Moderate (65% –80%), n (%)	High (≥80%), n (%)	Score range	Total score, mean (SD)	Average score (1–5), mean (SD)
Perceived usefulness	41 (12.7)	94 (29.2)	187 (58.1)	6–30	26.09 (4.09)	4.35 (0.68)
Perceived Ease of Use	181 (56.2)	81 (25.2)	60 (18.6)	6–30	21.64 (4.53)	3.61 (0.75)
Intention to use	79 (24.5)	112 (34.8)	131 (40.7)	4–20	16.22 (2.98)	4.05 (0.74)
Overall	81 (25.2)	129 (40.1)	112 (34.8)	16–80	63.95 (9.96)	4.0 (0.62)

**Table 4.** Relation between total score for overall (Artificial Intelligence Readiness, Technology acceptance) with demographic data (n=322).

Demographic data	n	Artificial Intelligence readiness, mean (SD)	Technology acceptance, mean (SD)
Age (years)			
18‐20	154	15.45 (2.27)	61.80 (9.08)
20‐22	119	16.33 (2.57)	65.34 (10.27)
>22	49	16.84 (2.62)	67.37 (10.49)
F^a^ (*P*)^b^		7.952^c^ (<.001^c^)	7.952^c^ (<.001^c^)
Sex			
Male	152	15.55 (2.55)	62.21 (10.21)
Female	170	16.38 (2.38)	65.51 (9.50)
t^d^ (*P*)		3.005^c^ (.003^c^)	3.005^c^ (.003^c^)
Academic level			
First-year	27	15.15 (2.61)	60.59 (10.44)
Second year	133	15.59 (2.30)	62.37 (9.20)
Third year	103	16.69 (2.51)	66.77 (10.04)
Fourth year	59	16.04 (2.57)	64.15 (10.29)
F (*P*)		5.077^c^ (.002^c^)	5.077^c^ (.002^c^)
Specialty			
Maternal and child nursing	26	16.30 (2.35)	65.19 (9.41)
Psychiatric Nursing	14	15.93 (2.80)	63.71 (11.21)
Community Nursing	26	14.38 (1.98)	57.50 (7.93)
Critical nursing	24	15.02 (2.86)	60.08 (11.44)
Administrative nursing	150	16.74 (2.19)	66.95 (8.78)
Adult nursing	82	15.33 (2.57)	61.30 (10.29)
F (*P*)		7.552^c^ (<.001^c^)	7.552^c^ (<.001^c^)

a F: F for One way ANOVA test

b *P*: *P* value for comparison between the studied categories

c Statistically significant at *P*≤.05

d t:Student *t*-test

## Discussion

### Principal Findlings

This study examined the readiness and acceptance of nursing students in Saudi Arabia regarding the use of artificial intelligence-based health care technology for nursing skills training. The findings indicate that most students are optimistic about incorporating AI into their education, with 69.6% reporting moderate readiness to adopt AI-related technological interventions. These results align with previous research showing that nurses generally hold positive attitudes and intentions toward the utilization of AI technologies in their practice and education [14,18]. Therefore, it is essential to incorporate specific courses on AI, promote student engagement with emerging technologies, and encourage faculty members to stay current with advancements in AI and its applications in nursing care [22].

Moreover, the study demonstrated that more than half of the participants (59% [190/322] and 59.3% [191/322]) experienced moderate discomfort and insecurity regarding the use of AI technologies. This highlights the need for increased efforts to educate nursing students about the efficacy and value of AI in training. In addition, participants demonstrated moderate techno-readiness toward adopting AI technologies. Sensitization initiatives should emphasize the benefits of AI, such as improving patient safety, enhancing the quality of clinical decision-making, and increasing efficiency in healthcare processes, including patient monitoring [15]. Furthermore, the moderate levels of techno-readiness highlight the need to increase nursing students’ exposure to technology in order to enhance their attitudes and perceptions of its value in nursing practice.

The overall technology acceptance among participants indicated that the majority exhibited moderate to high levels of acceptance. The findings reflect generally favorable attitudes toward the usefulness of technology, with more variability in perceived ease of use and intention to adopt it. While 58.1% of participants showed high acceptance regarding the perceived usefulness of technology, a notable proportion reported moderate to low acceptance. These findings highlight the need to foster greater engagement and raise awareness about the benefits and value of integrating technology into nursing education. As noted by Swan [14], such awareness is vital for facilitating positive shifts in perceptions and attitudes [22,23]. Additionally, perceived usefulness and ease of use of technological interventions, such as AI, have been consistently identified as strong predictors of willingness to adopt these technologies in nursing practice [14,17,20]. The results further indicate that most participants perceive the ease of use of AI technology as moderate to high. These findings are consistent with a study conducted in Nepal, which reported their moderate levels of digital literacy among nursing students [24]. However, they contrast with the findings of Stellesfson et al [25], who observed insufficient e-health literacy among college learners. This insufficiency is largely attributed to current curricula that often do not adequately incorporate the use of technologies such as AI in nursing and clinical education.

Additionally, the results indicate that the vast majority of participants (99.4%) accepted the use of technology, with only a small percentage (0.6%) reporting no acceptance. This underscores the importance of integrating innovative curricula at the undergraduate level to enhance nursing students’ exposure to emerging technologies such as AI. Early and structured exposure is crucial for building technological competency and fostering positive attitudes towards the use of AI in clinical practice [15]. Moreover, the study found no statistically significant differences in technology readiness and acceptance based on demographic variables such as age, gender, academic level, and specialty. This suggests that training programs should adopt a universal approach, targeting all nursing students equally. Such an inclusive strategy simplifies implementation, enhances cost-effectiveness, and supports the use of varied teaching methods to promote AI literacy. Educators can incorporate various methods such as role-plays, debates, panel discussions, and hands-on activities to enhance understanding of AI’s value and applications.

Furthermore, increasing students’ clinical exposure to AI technologies may positively shape their perceptions and encourage proactive engagement. Such shifts in perception are vital for fostering continuous learning and the development of essential nursing competencies in an AI-integrated environment. Nonetheless, several limitations should be acknowledged. First, the cross-sectional design restricts the ability to detect causal relationship between variables, making it challenging to determine the directionality of these correlations and understand how they might evolve over time. Second, the use of self-reported measures may introduce bias, as participants might provide socially desirable answers or may not accurately recall their experiences. Furthermore, while online survey distribution offers convenience, it may inadvertently exclude certain student groups, particularly those with limited access to or familiarity with digital platforms, thus potentially affecting the representativeness of the sample.

### Conclusion

The findings indicate that nursing students demonstrated a generally positive readiness and acceptance regarding the use of AI and technology in education and training. However, this readiness and acceptance were at a moderate level, highlighting the need for enhanced awareness and a deeper understanding of AI’s potential to improve training efficiency and the quality of healthcare delivery. To address this gap, nursing education programs should integrate AI-related content into their curricula, thereby fostering students’ familiarity, competence, and confidence in using emerging technologies in clinical and educational contexts.

## Abbreviations

AI: artificial intelligence
TAM: Technology Acceptance Model
TRI: technology readiness index
